# Autoimmune hemolytic anemia after allogeneic hematopoietic stem cell transplantation in adults: A southern China multicenter experience

**DOI:** 10.1002/cam4.2539

**Published:** 2019-09-10

**Authors:** Weiran Lv, Hong Qu, Meiqing Wu, Zhiping Fan, Fen Huang, Na Xu, Li Xuan, Ren Lin, Ke Zhao, Jing Sun, Yongrong Lai, Yajing Xu, Qifa Liu

**Affiliations:** ^1^ Department of Hematology Nanfang Hospital Southern Medical University Guangzhou Guangdong China; ^2^ Hematology Guangzhou Panyu Central Hospital Guangzhou Guangdong China; ^3^ Hematology The First Affiliated Hospital of Guangxi Medical University Nanning Guangxi China; ^4^ Hematology Xiangya Hospital Central South University Changsha Hunan China

**Keywords:** autoimmune hemolytic anemia, hematopoietic stem cell transplantation, risk factors, treatment

## Abstract

To investigate the incidence and risk factors as well as prognosis of autoimmune hemolytic anemia (AIHA) following allogeneic hematopoietic stem cell transplantation (allo‐HSCT), a total of 1377 adult hematological malignancies at three institutions were enrolled in this study. The 3‐year cumulative incidence of AIHA was 2.2 ± 0.4%. Multivariate analysis showed that haploidentical donors (HRDs) and chronic graft vs host disease (cGVHD) were the independent risk factors for AIHA. Patients with AIHA treated initially with corticosteroids combined with cyclosporine A (CsA) had a higher complete response rate than those with corticosteroids monotherapy (66.7% vs 11.1%; *P* = .013). The 3‐year cumulative incidence of malignant diseases relapse was 4.4 ± 4.3% and 28.0 ± 1.3% (*P* = .013), treatment‐related mortality (TRM) was 8.9 ± 6.3% and 17.4 ± 1.2% (*P* = .431), disease‐free survival (DFS) was 56.1 ± 1.5% and 86.7 ± 7.2% (*P* = .011), and overall survival (OS) was 86.3 ± 7.4% and 64.1 ± 1.5% (*P* = .054), respectively, in the patients with AIHA and those without AIHA. Our results indicate that HRDs and cGVHD are risk factors for AIHA and corticosteroids combined with CsA are superior to corticosteroids as initial treatment for AIHA. Autoimmune hemolytic anemia does not contribute to increase TRM and could reduce the malignant diseases relapse and increase DFS.

## Graphical abstract

Autoimmune hemolytic anemia (AIHA) occurs more frequently than other autoimmune hematological diseases (AHDs) after allogeneic hematopoietic stem cell transplantation. Haploidentical donors and chronic graft vs host disease are risk factors for AIHA and corticosteroids combined with cyclosporine A are superior to corticosteroids as initial treatment for AIHA. AIHA does not contribute to increase treatment‐related mortality and could reduce the malignant diseases relapse and increase disease‐free survival.

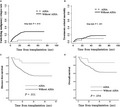

## 1 INTRODUCTION

Autoimmune hematological diseases (AHDs) have been reported to occur more frequently than other autoimmune complications after allogeneic hematopoietic stem cell transplantation (allo‐HSCT).^1^, ^2^, ^3^, ^4^, ^5^, ^6^, ^7^, ^8^ Autoimmune hematological diseases may affect a single lineage of blood cells, for example, autoimmune hemolytic anemia (AIHA) and immune thrombocytopenia (ITP), or 2 and/or 3 lineages, for example, Evans syndrome. In these AHDs, AIHA is the most common with estimates of the incidence between 2% and 6%^7^, ^9^, ^10^, ^11^ in recipients of allo‐HSCT. AIHA posttransplants have been proved to be associated with many factors, such as human leukocyte antigen (HLA)‐mismatched transplants, chronic graft vs host disease (cGVHD), using antithymocyte globulin (ATG), and so on.^5^, ^12^, ^13^, ^14^ In recent years, an increasing incidence of AIHA has been observed. Haploidentical and unrelated transplants have been widely used,^12^, ^15^, ^16^ whether haploidentical transplants could cause AIHA is rarely reported. Corticosteroids are usually used as first‐line treatment for AIHA, but the effective rate is approximately 10%‐40% in patients with AIHA posttransplants.^3^, ^10^, ^17^, ^18^ In our multicenter report, we retrospectively analyzed the incidence and risk factors, and the outcomes of corticosteroids combined with cyclosporine A (CsA) or corticosteroids monotherapy as initial treatment in the patients developed AIHA after allo‐HSCT.

## 2 PATIENTS AND METHODS

### 2.1 Study design and patients

All consecutive adult patients with hematological malignancies who underwent first allo‐HSCT between December 2011 and December 2016 at Nanfang Hospital, Xiangya Hospital, and First Affiliated Hospital of Guangxi Medical University were analyzed in this retrospective study. Medical records for all patients were reviewed for demographic data, primary diseases, transplant‐related parameters, and AIHA including information on the history and treatment of AIHA pretransplantation. If the patients had a history of AIHA pretransplantation, they were excluded. This study was performed in accordance with the Declaration of Helsinki and was approved by the Institution Review Board of our institution.

### 2.2 Transplant procedures

High‐resolution molecular techniques were used to detect HLA typing of recipients and donors.^11^ All patients received myeloablative conditioning regimens. The selection of conditioning regimens was as follows: (a) BuCY (busulfan + cyclophosphamide) or BuF (busulfan + Fludarabine) was applied to patients who had myeloid malignancies with complete remission (CR); (b) TBI + CY (total body irradiation + cyclophosphamide) were applied to patients who had lymphoid malignancies with CR; (c) fludarabine + cytarabine + TBI + CY + etoposide (VP‐16) were applied to patients with no CR (NR).^11^, ^16^, ^19^ Grafts from peripheral blood stem cells (PBSCs) were used in HLA‐matched sibling transplantation patients and unrelated donor transplantation patients. Grafts from PBSCs combined with bone marrow (BM) were used in haploidentical transplantation patients. The selection of graft vs host disease (GVHD) prophylaxis regimens was as follows: (a) CsA + methotrexate (MTX) (at days + 1, 3, and 6) were applied to patients who received transplants from HLA‐matched sibling donor (MSD); (b) CsA + MTX + ATG (7.5 mg/kg) were applied to patients who received transplants from matched unrelated donor (MUD); (c) CsA + MTX + ATG (7.5‐10 mg/kg) + mycophenolate mofetil (MMF) (0.5 g, 2/d × 28 days) were applied to patients who received transplants from haploidentical donor (HRD).^11^, ^16^

### 2.3 Diagnosis and response criteria of AIHA

As previously reported,^14^, ^18^, ^20^ the diagnostic criteria of AIHA and Evans syndrome diagnoses were described below. The diagnostic criteria of AIHA included: (a) a positive direct antiglobulin test (DAT); (b) a positive indirect antiglobulin test with broad reactivity to red blood cells in the serum and eluate; (c) clinical and laboratory evidence of hemolysis (increase in lactate dehydrogenase and bilirubin levels, decrease in hemoglobin [Hb] and haptoglobin levels, or increase in transfusion requirements); and (d) a differentiation diagnosis. Patients of DAT positivity caused by ABO (blood group of ABO) antibodies and patients had history of AIHA or a positive DAT before HSCT were excluded. Patients who never had a DAT positivity were not presumed to have clinically significant AIHA. Patients who had a positive DAT but had evidence of nonimmune hemolysis, for example microangiopathic hemolytic anemia, were also excluded. Furthermore, the primary and secondary poor graft function posttransplants were also excluded from the diagnosis of AIHA after allo‐HSCT. The diagnostic criteria of Evans syndrome: either a simultaneous combination or a sequential combination of ITP and AIHA with DAT positivity.

Responses were mainly evaluated at 4 and 12 weeks after initial treatment. Thresholds for determining response were based on standard and previous studied outcome criteria for AIHA and Evans syndrome.^11^, ^21^ The criteria for effectiveness were as follows: (a) CR: Hb level of 12 g/dL and a platelet (PLT) level of 100 g/L or more in the absence of a transfusion without features of hemolysis (normal bilirubin and lactate dehydrogenase levels ± normal haptoglobin level if performed); (b) partial response (PR): Hb level of at least 10 g/dL with an increase of at least 2 g from baseline and a PLT level of at least 50 g/L; (c) NR: failure to meet the above two criteria; and (d) the overall response (OR) rate included both CR and PR.

### 2.4 Evaluation points and definitions

This study mainly focused on the incidence and risk factors of AIHA, treatment response, treatment‐related mortality (TRM), malignant diseases relapse, disease‐free survival (DFS), and overall survival (OS). In patients with AIHA in CR or PR, relapse of AIHA was defined as the loss of CR or PR status, respectively. Relapse of malignant diseases was defined by reappearance of blasts in the peripheral blood, recurrence of BM blasts >5%, or development of extramedullary disease infiltrates at any site. Treatment‐related mortality was defined as death from any cause except malignant diseases relapse. Disease‐free survival was defined as survival in a state of continuous CR. Overall survival was defined as the earliest time from AIHA diagnosis to death from all causes.

### 2.5 Statistical analysis

Patient follow‐up was updated on December 2017. Data were presented as the mean ± SD or median (range) for continuous variables, depending on the distribution. Categorical variables were presented as numbers (%). The Chi‐squared or Fisher exact tests were used to compare proportions. Disease‐free survival and OS were analyzed with the Kaplan‐Meier method, comparing groups using the log rank test (Mantel‐Haenszel). Treatment‐related mortality and relapse were calculated using reciprocal cumulative incidence estimates to account for competing risks (Gray test). Risk factors achieving statistical significance upon univariate analysis underwent additional multivariate analysis using Cox regression to identify the most significant independent risk factors. All *P* values were two‐sided and considered significant with *P* < .05. Statistical analyses were performed with SPSS Version 19.0.

## 3 RESULTS

### 3.1 Patients' demographics and baseline characteristics

Of the 1381 patients with hematological malignancies enrolled in this retrospective study, 1377 were retained for analysis, and four were excluded due to the history of AIHA before transplantation. Among the 1377 patients retained for analysis, 651 patients came from Nanfang Hospital, 266 patients came from Xiangya Hospital, and 460 patients came from First Affiliated Hospital of Guangxi Medical University. These patients had a median age of 30 years (range, 13‐78 years), with 752 males and 625 females. The underlying diseases included myelogenous leukemia (n = 782) and lymphoid leukemia (n = 595). Nine hundred and ninety‐three patients achieved CR and 384 patients were in PR or NR at the time of transplantation. Seven hundred and sixty‐six patients received MSD, 328 MUD, and 283 HRD transplants.

### 3.2 Incidence and risk factors of AIHA

Twenty‐six patients had AIHA, including 19 with AIHA and seven with AIHA accompanied with thrombocytopenia (Evans syndrome). Eleven were females and 15 males with a median age of 23.5 years (range, 15‐46) at transplants. The median time of AIHA onset was 215 days (range, 34‐756 days) posttransplants. The median number of white blood cell count, Hb count, and PLT count at the time of AIHA diagnosis was 3.13 G/L (range, 1.32‐7.23), 57.5 g/L (range, 34‐75), and 133 G/L (range, 11‐187), respectively. These patients all had complete donor chimerism at time of diagnosis of AIHA. At the time of AIHA onset, 15 patients were treated with immunosuppressive agents, including six cases with GVHD prophylaxis that gradually tapered and nine with GVHD treatment (Table 1). The baseline and transplant characteristics of patients with and without AIHA are shown in Table 2.

**Table 1:** Characteristics of patients with AIHA

No	Gender	Age	Diagnosis	Donor type	Type of AIHA	Time to AIHA	cGVHD	Immunosuppressive therapies at the onset of AIHA	Initial treatment	AIHA relapse	Underlying disease relapse
1	Male	46	AML	HRD	Evans	245	Yes	MMF(0.25 g2/d) + tacrolimus (0.5 mg 2/d)	GC + CsA	No	No
2	Male	21	MDS	HRD	Evans	203	Yes	MMF(0.25 g 2/d) + CsA (150 mg 1/d) + methylprednisolone (32 mg 1/d)	GC + CsA	No	No
3	Male	18	T‐LBL	MUD	AIHA	312	No	CsA (50 mg 2/d) + methylprednisolone (32 mg 1/d)	GC + CsA	No	No
4	Male	25	AML	HRD	Evans	133	Yes	CsA (50 mg 2/d) + methylprednisolone (20 mg 1/d)	GC + CsA	No	No
5	Male	37	ALL	HRD	AIHA	189	No	Methylprednisolone (60 mg 1/d)	—a	—a	No
6	Male	38	AUL	MSD	AIHA	375	Yes	Methylprednisolone (40 mg 1/d) + prograf (1.5 mg 2/d)	GC + CsA	—b	No
7	Female	29	ALL	MSD	AIHA	756	No	methylprednisolone(32 mg 1/d)	GC + CsA	No	No
8	Female	46	MDS	HRD	Evans	60	No	MMF (0.5 g 2/d) + CsA (50 mg 2/d) + methylprednisolone (40 mg 3/d)	GC + CsA	No	No
9	Female	20	ALL	HRD	AIHA	140	No	MMF (0.25 g 1/d) + CsA (50 mg 2/d) + methylprednisolone (40 mg 1/d)	GC + CsA	—b	No
10	Male	18	ALL	HRD	Evans	104	No	MMF (0.2 g 2/d) + tacrolimus (0.5 mg 2/d) + methylprednisolone (40 mg 1/d)	GC + CsA	—b	No
11	Male	16	ALL	MUD	AIHA	154	Yes	CsA (50 mg 2/d) + methylprednisolone (32 mg 1/d)	GC + CsA	No	No
12	Male	19	ALL	HRD	Evans	203	No	MMF(0.5 g 2/d) + CsA (50 mg 2/d) + methylprednisolone (40 mg 3/d)	GC + CsA	No	No
13	Male	22	ALL	MUD	AIHA	252	Yes	—	GC + CsA	No	No
14	Male	22	AML	HRD	AIHA	215	Yes	—	GC	No	No
15	Male	44	AML	MUD	Evans	111	Yes	CsA (25 mg 2/d)	GC	Yes	No
16	Male	44	ALL	MSD	AIHA	258	Yes	—	GC	—b	No
17	Female	15	ALL	HRD	AIHA	34	No	MMF (0.25 g 2/d) + tacrolimus (0.5 mg 2/d)	GC + CsA	No	No
18	Female	36	AML	HRD	AIHA	134	Yes	MMF (0.25 g 2/d) + CsA (150 mg 1/d) + methylprednisolone (32 mg 1/d)	GC	Yes	No
19	Female	31	ALL	HRD	AIHA	139	Yes	CsA (50 mg 2/d) + methylprednisolone (32 mg 1/d)	GC	—b	No
20	Male	20	ALL	MSD	AIHA	254	Yes	—	GC	Yes	No
21	Female	27	ALL	HRD	AIHA	349	Yes	—	GC	Yes	No
22	Female	26	ALL	HRD	AIHA	263	Yes	—	GC	—c	No
23	Male	18	AML	HRD	AIHA	250	Yes	—	GC	No	No
24	Female	22	AML	MUD	AIHA	220	Yes	MMF (0.5 g 2/d) + CsA (50 mg 2/d) + methylprednisolone (40 mg 3/d)	GC + CsA	No	No
25	Female	34	AML	MSD	AIHA	197	No	—	GC + CsA	No	No
26	Female	21	AML	MUD	AIHA	228	Yes	—	GC	No	Yes

Abbreviations: AIHA, autoimmune hematological diseases; ALL, acute lymphocytic leukemia; AML, acute myeloid leukemia; AUL, acute undifferentiated leukemia; CsA, cyclosporine A; GC, glucocorticoid; HRD, haploidentical‐related donor; MDS, myelodysplastic syndrome; MMF, mycophenolate mofetil; MSD, matched sibling donor; MUD, matched unrelated donor; T‐LBL, T‐lymphoblastic lymphoma.

a This patient died of infectious shock 2 days after the diagnosis of AIHA and was excluded from AIHA treatment and relapse analyses.

b These patients received corticosteroids combined CsA treatment who obtained no response after the 4 weeks' initial treatment changed their regimen by adding second‐line treatments so that was excluded from AIHA relapse analyses.

c This patient received corticosteroids monotherapy who did not response to the 2 weeks' initial treatment changed their regimen by adding CsA so that was excluded from AIHA relapse analyses.

**Table 2:** Characteristics of patients with and without AIHA

Characteristic	Patients with AIHA	Patients without AIHA	*P*
Gender, n (%)			.457
Male	15 (58)	737 (55)	
Female	11 (42)	614 (45)	
Median age at HSCT, y (range)	23.5 (15‐46)	30 (13‐78)	.466
Type of underlying disease, n (%)			.318
Myelogenous	12(46)	770 (57)	
Lymphoid	14 (54)	581 (43)	
Disease status at HSCT, n (%)			.741
CR	18 (69)	975 (72)	
Non‐CR	8 (31)	376 (28)	
Donor source, n (%)			<.001*
MSD	6 (23)	760 (56)	
MUD	5 (19)	323 (24)	
HRD	15 (58)	268 (20)	
HLA disparity, n (%)			<.001*
Matched	11 (42)	1045 (77)	
Mismatched	15 (58)	306 (23)	
ABO matched, n (%)			.985
Yes	13 (50)	678 (50)	
No	13 (50)	673 (50)	
Sex matched, n (%)			.232
Yes	15 (58)	620 (46)	
No	11 (42)	731 (54)	
Conditioning regimens, n (%)			.443
TBI used	17 (65)	782 (58)	
TBI non‐used	9 (35)	569 (42)	
GVHD prophylaxis, n (%)			.001*
ATG used	21 (81)	651 (48)	
ATG non‐used	5 (19)	700 (52)	
Source of stem cell, n (%)			<.001*
Bone marrow + PBSCs	15 (58)	294 (22)	
PBSCs	11 (42)	1057 (78)	
CMV viremia posttransplants, n (%)			.991
Yes	14 (54)	726 (54)	
No	12 (46)	625 (46)	
aGVHD, n (%)			.711
Yes	11 (42)	621 (46)	
No	15 (58)	730 (54)	
cGVHD, n (%)			.009*
Yes	18 (69)	582 (43)	
No	8 (31)	769 (57)	

Abbreviations: aGVHD, acute graft vs host disease; AIHA, autoimmune hematological diseases; ATG, antithymocyte globulin; cGVHD, chronic graft vs host disease; CMV, cytomegalovirus; CR, complete remission; GVHD, graft vs host disease; HLA, human leukocyte antigen; HRD, haploidentical‐related donor; HSCT, hematopoietic stem cell transplantation; MSD, matched sibling donor; MUD, matched unrelated donor; TBI, total body irradiation; PBSCs, peripheral blood stem cells.

* *P* < .05.

**Figure 1:**
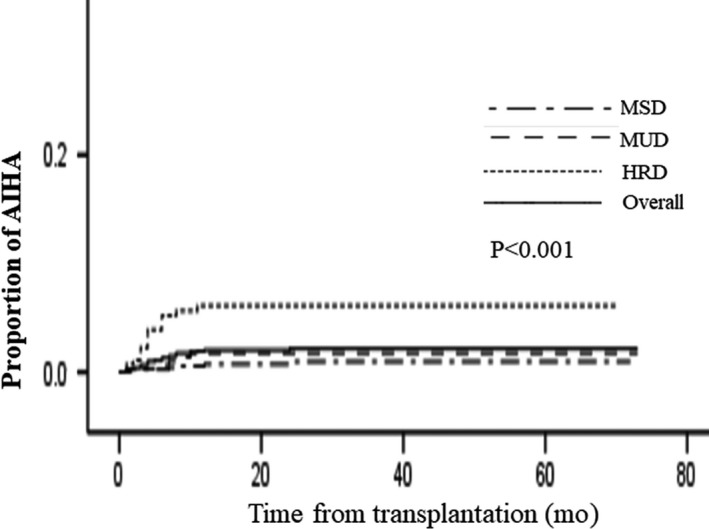
Cumulative incidence of autoimmune hemolytic anemia (AIHA) according to type of donor

The overall 3‐year incidence of AIHA posttransplantation was 2.2 ± 0.4%, and the 3‐year incidence of AIHA in HRD, MUD, and MSD was 6.3 ± 1.6%, 1.8 ± 0.8% and 1.0 ± 0.4%, respectively. HRD had higher incidence than MUD (*P* < .001) and MSD (*P* = .004), but there was no difference between MUD and MSD transplants (*P* = .222) (Figure 1). Univariate analysis showed that donor source, HLA mismatched, ATG, and cGVHD were risk factors for AIHA (Table 2), but multivariate analysis showed that only HRD and cGVHD were risk factors for AIHA (Table 3).

**Table 3:** Multivariate analysis for risk factors of AIHA

Variable	Multivariate (HR)
Male vs female	*P* = .860
Patient age, >30 y old, ≤30 y old	*P* = .956
Myelogenous vs lymphoid	*P* = .079
MSD vs HRD	*P* < .001 (7.076) 95% CI: 2.741‐18.265
MUD vs HRD	*P* = .012 (3.679) 95% CI: 1.336‐10.132
MSD vs MUD	*P* = .276
CR vs non‐CR	*P* = .576
PBSCs vs PBSCs + BM	*P* = .957
HLA matched vs mismatched	*P* = .802
ABO matched vs mismatched	*P* = .667
Sex matched vs mismatched	*P* = .374
ATG used vs non‐used	*P* = .332
TBI used vs non‐used	*P* = .297
CMV viremia positive vs negative	*P* = .581
aGVHD vs non‐aGVHD	*P* = .468
cGVHD vs non‐cGVHD	*P* = .028 (2.554) 95% CI: 1.109‐5.884

Abbreviations: aGVHD, acute graft vs host disease; AIHA, autoimmune hematological diseases; ATG, antithymocyte globulin; BM, bone marrow; cGVHD, chronic graft vs host disease; CI, confidence interval; CMV, cytomegalovirus; CR, complete remission; HLA, human leukocyte antigen; HR, hazard ratio; HRD, haploidentical‐related donor; MSD, matched sibling donor; MUD, matched unrelated donor; PBSCs, peripheral blood stem cells; TBI, total body irradiation.

### 3.3 Treatment and response

Among the 26 patients diagnosed AIHA, only 25 patients had access to treatment because one patient with AIHA died of infectious shock 2 days after the diagnosis of AIHA. Of the 25 patients who received treatment on the basis of their original immunosuppressive agents, 15 had corticosteroids (1‐2 mg/kg) combined with CsA as initial treatment and the remaining 10 patients had corticosteroids (1‐2 mg/kg) monotherapy as initial treatment. Baseline information between the two treatment groups is shown in Table 4.

**Table 4:** Baseline information between the two treatment groups

	CsA + GC	GC	*P*‐value
Number	15	10	
Age (y) Median (range)	22 (15‐46)	26.5 (18‐44)	.478
Disease type Myelogenous/Lymphoid	6/9	5/5	.697
Transplant type HRD/MSD/MUD	8/3/4	6/2/2	.924
Sex ratio (men/women)	9/6	5/5	.466
Pretransplant disease state CR/Non‐CR	11/4	7/3	1.000
Stem cell source PBSC/PBSC + BM	7/8	4/6	.742
HLA matched Yes/No	7/8	4/6	.742
ABO matched Yes/No	8/7	5/5	1.000
Sex matched Yes/No	7/8	7/3	.414
ATG/CD25 used Yes/No	11/4	9/1	.615
TBI used Yes/No	10/5	6/4	.734
CMV viremia Positive/Negative	10/5	3/7	.111
aGVHD Yes/No	7/8	4/6	.742
cGVHD Yes/No	7/8	10/0	.008
AHDs type AIHA/Evans	9/6	9/1	.179

Abbreviations: aGVHD, acute graft vs host disease; AHDs, autoimmune hematological diseases; AIHA, autoimmune hemolytic anemia; ATG, antithymocyte globulin; BM, bone marrow; cGVHD, chronic graft vs host disease; CMV, cytomegalovirus; CsA, cyclosporine A; CR, complete remission; Evans, Evans syndrome; GC, glucocorticoid; HLA, human leukocyte antigen; HRD, haploidentical‐related donor; MSD, matched sibling donor; MUD, matched unrelated donor; PBSCs, peripheral blood stem cells; TBI, total body irradiation.

After 4 weeks of initial treatment, one patient who received corticosteroids treatment was excluded from effect analysis because the patient did not respond to the 2‐week initial corticosteroids treatment and received corticosteroids combined with CsA. The OR rate was 80.0% and 77.7% (*P* = .635), and the CR rate was 66.7% and 11.1% (*P* = .013), respectively, in patients who received corticosteroids combined with CsA and corticosteroids monotherapy. Five patients who had no response to treatment after 4 weeks all received second‐line treatments, including rituximab (n = 4) and CsA + MMF (n = 1). Within 12 weeks of treatment, all patients had response. One patient (6.7%) experienced AIHA relapse who received corticosteroids combined with CsA treatment, while five (50.0%) experienced relapse who received corticosteroids monotherapy (*P* = .023) at a median follow‐up of 22 months (range, 6‐56 months). Fortunately, all of the relapsed patients achieved remission again after immunosuppressive therapy.

### 3.4 Survival

**Figure 2:**
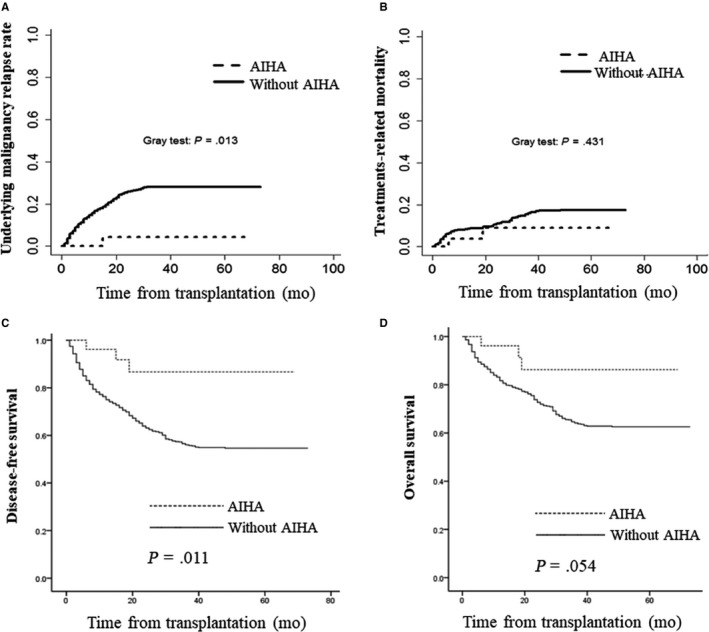
Relapse, treatment‐related mortality, disease‐free survival, and overall survival in patients with and without autoimmune hematological diseases (AIHA). A, Accumulation underlying malignancy relapse rate in patients with and without AIHA. B, Accumulation of treatment‐related mortality in patients with and without AIHA. C, Accumulation of disease‐free survival function in patients with and without AIHA. D, Accumulation of survival function in patients with and without AIHA

Twenty‐three patients were alive and three were dead at a median follow‐up of 662 days (range, 2‐1726 days) after AIHA. The causes of death included infections (n = 2) and leukemia relapse (n = 1). To avoid the effect of the patients who died or relapsed before onset of AIHA, we chose the earliest date of AIHA onset (on 34 day posttransplants) as a landmark to calculate the outcome (relapse, OS, TRM). Of the 1351 patients without AIHA, 1335 cases were enrolled in analysis for malignant disease relapse, TRM, DFS, and OS based on the above contents. The 3‐year cumulative incidence of malignant diseases relapse, DFS, and OS posttransplant were 27.5 ± 1.2%, 56.1 ± 1.5%, and 64.5 ± 1.5%, respectively. The 3‐year cumulative incidence of malignant diseases relapse was 4.4 ± 4.3% and 28.0 ± 1.3% (*P* = .013) (Figure 2A), TRM posttransplants was 8.9 ± 6.3% and 17.4 ± 1.2% (*P* = .431) (Figure 2B), DFS was 56.1 ± 1.5% and 86.7 ± 7.2% (*P* = .011) (Figure 2C), and OS was 86.3 ± 7.4% and 64.1 ± 1.5% (*P* = .054) (Figure 2D), respectively, for patients with AIHA and those without AIHA. Risk factors for malignant diseases relapse and survival are presented in Table 5. In multivariate analysis for malignant diseases relapse and DFS, myelogenous disease, CR at transplantation, cGVHD, and AIHA were beneficial factors. In multivariate analysis for OS, CR at transplantation and cGVHD were beneficial factors.

**Table 5:** Univariate and multivariate analyses for malignant diseases relapse, DFS, and OS

Variable	Relapse		DFS		OS	
	Univariable	Multivariable (HR)	Univariable	Multivariable (HR)	Univariable	Multivariable (HR)
Male vs female	*P* = .773	*P* = .925	*P* = .747	*P* = .727	*P* = .422	*P* = .475
Patient age, >30 y old, ≤30 y old	*P* = .300	*P* = .965	*P* = .770	*P* = .649	*P* = .511	*P* = .512
Myelogenous vs lymphoid	*P* = .005	*P* = .004 (0.732) 95% CI: 0.592‐0.904	*P* = .086	*P* = .026 (0.824) 95% CI: 0.694‐0.978	*P* = .667	*P* = .295
Donor type	*P* = .041		*P* = .021		*P* = .080	
MSD vs HRD		*P* = .365		*P* = .431		*P* = .264
MUD vs HRD		*P* = .113		*P* = .733		*P* = .147
MSD vs MUD		*P* = .312		*P* = .778		*P* = .556
CR vs non‐CR	*P* < .001	*P* < .001 (0.668) 95% CI: 0.536‐0.833	*P* < .001	*P* = .004 (0.765) 95% CI: 0.639‐0.916	*P* < .001	*P* < .001 (0.678) 95% CI: 0.554‐0.829
PBSCs vs PBSCs + BM	*P* = .062	*P* = .531	*P* = .022	*P* = .584	*P* = .047	*P* = 309
HLA matched vs mismatched	*P* = .016	*P* = .263	*P* = .010	*P* = .411	*P* = .032	*P* = 242
ABO matched vs mismatched	*P* = .817	*P* = .651	*P* = .911	*P* = .713	*P* = .745	*P* = 911
Sex matched vs mismatched	*P* = .216	*P* = .136	*P* = .250	*P* = .159	*P* = .337	*P* = 227
ATG used vs non‐used	*P* = .705	*P* = .958	*P* = .263	*P* = .706	*P* = .083	*P* = .190
TBI uses vs non‐used	*P* < .001	*P* = .798	*P* = .001	*P* = .795	*P* < .001	*P* = .214
CMV viremia positive vs negative	*P* = .341	*P* = .484	*P* = .880	*P* = .618	*P* = .068	*P* = .444
aGVHD vs non‐aGVHD	*P* = .186	*P* = .131	*P* = .989	*P* = .432	*P* = .361	*P* = .849
cGVHD vs non‐cGVHD	*P* < .001	*P* < .001 (0.667) 95% CI: 0.532‐0.838	*P* < .001	*P* = .001 (0.738) 95% CI: 0.617‐0.883	*P* < .001	*P* < .001 (0.666) 95% CI: 0.540‐0.821
AIHA vs non‐AIHA	*P* = .011	*P* = .049 (0.139) 95% CI: 0.020‐0.999	*P* = .003	*P* = .026 (0.275) 95% CI: 0.088‐0.856	*P* = .036	*P* = .074

Abbreviations: aGVHD, acute graft vs host disease; AIHA, autoimmune hematological diseases; ATG, antithymocyte globulin; BM, bone marrow; cGVHD, chronic graft vs host disease; CI, confidence interval; CMV, cytomegalovirus; CR, complete remission; DFS, disease‐free survival; HLA, human leukocyte antigen; HR, hazard ratio; HRD, haploidentical‐related donor; MSD, matched sibling donor; MUD, matched unrelated donor; OS, overall survival; PBSCs, peripheral blood stem cells; TBI, total body irradiation.

## 4 DISCUSSION

In this study, we retrospectively reviewed the incidence of AIHA in a multicenter from southern China. Our result showed that the 3‐year incidence of AIHA in HRD, MUD, and MSD was 6.3 ± 1.6%, 1.8 ± 0.8%, and 1.0 ± 0.4%, respectively, which was consistent with our single‐center report.^11^ Multivariate analysis demonstrated that HRD and cGVHD were risk factors of AIHA posttransplants. It has been reported that the most common AHDs posttransplant was AIHA.^7^, ^10^, ^13^, ^14^ In our group of 26 patients with AIHA, our result showed that seven patients diagnosed with Evans syndrome were accompanied with thrombocytopenia and they mainly occurred in HRD patients (n = 6).

The treatment of AIHA posttransplants is no consensus. This AIHA is more refractory to corticosteroids as a first‐line treatment compared with primary AIHA.^18^, ^22^, ^23^ To those who failed in response to corticosteroids treatment, second‐ and third‐line treatments, such as rituximab, CsA, and MMF, were administered, with an effective rate of approximately 60%‐85%.^3^, ^10^, ^17^, ^18^ In this report, we compared the efficacy of corticosteroids combined with CsA to corticosteroids monotherapy as initial treatment for AIHA after allo‐HSCT. Our results demonstrated that corticosteroids combined with CsA were superior to corticosteroids monotherapy as initial treatment for AIHA. Rituximab is frequently used as second‐line therapy for AIHA, with effective rate of above 80%.^7^, ^10^, ^24^, ^25^ In our study, the four patients who failed to respond to initial therapy all had a response to rituximab. Relapse occurred in approximately 50% of patients with primary AIHA, but the relapse rate for AIHA posttransplants had rarely been reported.^26^, ^27^ In our study, the relapse rate was higher in the patients received corticosteroids monotherapy compared with those received corticosteroids combined with CsA treatment. Whether AIHA contributes to increase TRM has not yet been defined.^7^, ^12^, ^18^ Daikeler et al suggested that AIHA was not attributable to TRM.^7^, ^12^ In contrast, Sokol et al^28^ and Meng et al^18^ reported that AIHA contributed to increase TRM. Our results demonstrated that AIHA did not contribute to increase mortality instead of increasing DFS. The good survival of AIHA patients was attributed to good therapeutic responses of AIHA and a lower rate of relapse for primary malignancies. Interestingly, only one patient experienced primary malignancy relapse in 26 patients with AIHA in our study and the 3‐year cumulative incidence of malignant diseases relapse was 4.4 ± 4.3% while the incidence of patients without AIHA was 28.0 ± 1.3% (*P* = .013). Our result showed that patients with AIHA had low primary malignancy relapse which was consistent with Sanz J's study.^14^ Sanz reported that none of the 12 cases with AIHA died of primary malignancy relapse. A reasonable interpretation of low primary malignancy relapse rate in patients with AIHA might be grafts vs malignancy (GVM) effects in these patients.^29^, ^30^ In this report, our result showed that cGVHD was a risk factor of AIHA and it was a protective factor for primary malignancy relapse, which supported our above interpretation. Whether AIHA‐induced immune responses contributed to GVM effects is worth further study.

A major limitation of the study was that this is a retrospective analysis of data. Some relevant factors might not be deeply discussed and found because of incomplete data records and the data records might not be accurate enough which could increase the error of the acquired data.

## 5 CONCLUSION

In conclusion, our results suggest that HRD transplants and cGVHD are risk factors for AIHA and corticosteroids combined with CsA are superior to corticosteroids as initial treatment for AIHA. Notably, AIHA does not contribute to increase TRM instead of contributing to reduce primary diseases relapse.

## CONFLICT OF INTEREST

None declared.

## AUTHOR CONTRIBUTIONS

W.‐R.L., H.Q., and M.‐Q.W. performed investigations, analyzed data, and wrote the paper; Z.‐P.F. and F.H. analyzed data; N.X., L.X., R.L., K.Z., and J.S. performed investigations; Y.‐R.L, Y.‐J.X., and Q.‐F.L. designed study. All authors read and approved the final manuscript.
